# The Efficient Synthesis and Anti-Fatigue Activity Evaluation of Macamides: The Unique Bioactive Compounds in Maca

**DOI:** 10.3390/molecules28093943

**Published:** 2023-05-07

**Authors:** Tao Liu, Ziyan Peng, Wei Lai, Yan Shao, Qing Gao, Miaoxin He, Wan Zhou, Lirong Guo, Jiyao Kang, Xiaobao Jin, Hui Yin

**Affiliations:** 1 Guangdong Provincial Key Laboratory of Pharmaceutical Bioactive Substances, School of Basic Medical Sciences, Guangdong Pharmaceutical University, Guangzhou 510006, China; liutao@gdpu.edu.cn (T.L.); 15812315359@163.com (Z.P.); laiwei1227@foxmail.com (W.L.); 2112240280@gdpu.edu.cn (M.H.);; 2 Department of Microbiology and Immunology, Guangdong Pharmaceutical University, Guangzhou 510006, China; 3 School of Pharmacy, Guangdong Pharmaceutical University, Guangzhou 510006, China; 4 National Key Laboratory of Biochemical Engineering, Institute of Process Engineering, Chinese Academy of Sciences, Beijing 100080, China; jykang@ipe.ac.cn

**Keywords:** macamides, bioactive marker compounds, synthesis, carbodiimide condensation method, anti-fatigue

## Abstract

Macamides are a class of amide alkaloids that are only found in maca and are widely considered to be its bioactive marker compounds. More than thirty macamide monomers have been identified in recent years; however, it is difficult to obtain a single macamide monomer from the maca plant because of their similar structures and characteristics. We used the carbodiimide condensation method (CCM) to efficiently synthesize five typical macamides, including *N-*benzyl-hexadecanamide (NBH), *N-*benzyl-9Z,12Z,15Z-octadecenamide, *N-*(3-methoxybenzyl)-9Z,12Z-octadecenamide, *N-*benzyl-9Z,12Z-octadecenamide, and *N-*(3-methoxybenzyl)-9Z,12Z,15Z-octadecadienamide. All the synthesized macamides were purified by a one-step HPLC with a purity of more than 95%. NBH is the most abundant macamide monomer in natural maca, and it was selected to evaluate the anti-fatigue effects of macamides. The results indicated that NBH could enhance the endurance capacity of mice by increasing liver glycogen levels and decreasing blood urea nitrogen, lactate dehydrogenase, blood ammonia, and blood lactic acid levels. Macamides might be the active substances that give maca its anti-fatigue active function.

## 1. Introduction

*Lepidium meyenii walp.* is an annual or biennial Andean crop, also known as maca in Peru, where it can be extensively grown at high altitudes (3500–4800 m). Its underground storage hypocotyls have been a traditional medicinal herb and dietary staple since pre-Columbian times [1]. The underground root of this plant is rich in macamides, maca alkaloids, glucosinolates, volatile oils, sterols, polyphenols, and macaenes [2,3]. A wide array of maca extracts has been obtained by petroleum ether [4], pentane [5], methanolic [6], alcoholic [7,8], or aqueous [9] extraction and widely used in pharmacological studies. Macamides, secondary amides with variable hydrocarbon chain lengths and levels of unsaturation, are formed by combining benzylamine and a fatty acid moiety. Macamides are considered functional components of the maca plant, unique to this species. Research in recent years has shown that macamides have a variety of pharmacological activities, including a vital effect on endurance capacity [10], neuroprotection [10,11], colitis [12], ovarian injury [13], and testicular function and spermatogenesis [14]. Moreover, some macamides act as irreversible inhibitors of fatty acid amide hydrolases (FAAH) and have anti-fatigue properties [15]. However, the macamides content, especially highly unsaturated macamides, in maca is low, and the extraction steps are more tedious, making it problematic to obtain abundant and pure single macamides. Therefore, to further validate the biological activity and mechanisms of macamides, high-purity macamides should be employed instead of maca extracts. However, due to their special physicochemical properties, macamides cannot be easily isolated from maca.

The amides of these macamides are derivatives of oleic, linoleic, and linolenic acids combined with either benzylamine or 3-methoxybenzylamine [16] and contain an amide bond in their structure. Thus, an investigation of amide-bond synthetic methods could be useful in acquiring the desired macamides. Conventional amide-synthetic methods include a nucleophilic substitution of carboxylic acids (and their derivatives) with amines. Due to its low activity, the carboxylic acid typically converts to an activated derivative and then reacts with an amine to generate an amide.

In this study, the synthesis of the five main macamides was investigated using the CCM. Moreover, optimization of their purification processes was examined to increase both the purity and yield of these macamides. Additionally, the optimal synthetic conditions for these five macamides should use inexpensive raw materials and provide high product purity and yield. The structures of these macamides were established by spectrometric and spectroscopic methods, including ESI-MS, IR, ^1^H NMR, and ^13^C NMR. Although maca extracts in various solvents have occasionally been found to have anti-fatigue properties, there has not been much research on the anti-fatigue properties of pure components. In this experiment, NBH was selected, and extracts of macamides were used as controls to assess the anti-fatigue capacity of the synthetic macamides.

## 2. Results

### 2.1. Synthesis, Purification and Identification of Macamides

**Figure 1:**
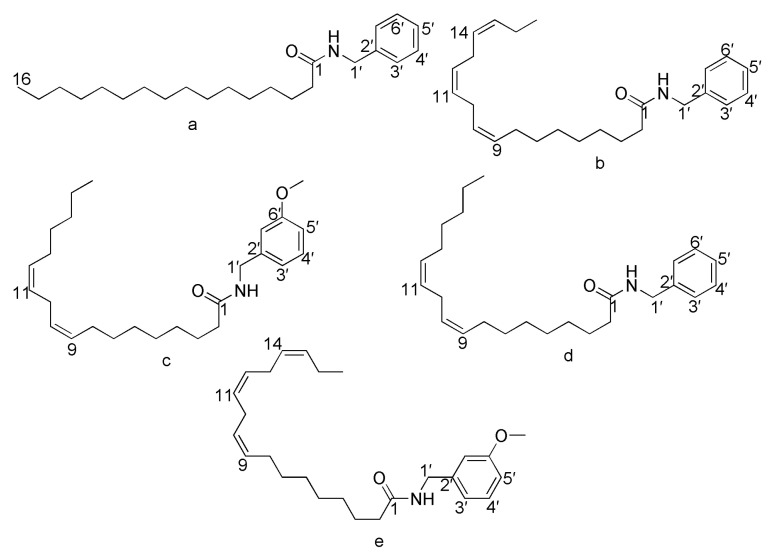
Structural formulas of five typical macamides. (**a**): N-benzyl-hexadecanamide (NBH), (**b**): N-benzyl-9Z,12Z,15Z-octadecatrienamide, (**c**): N-(3-methoxybenzyl)-9Z,12Z-octadecadienamide, (**d**): N-benzyl-9Z,12Z-octadecadienamide, (**e**): N-(3-methoxybenzyl)-9Z,12Z,15Z-octadecatrienamide.

**Figure 2:**
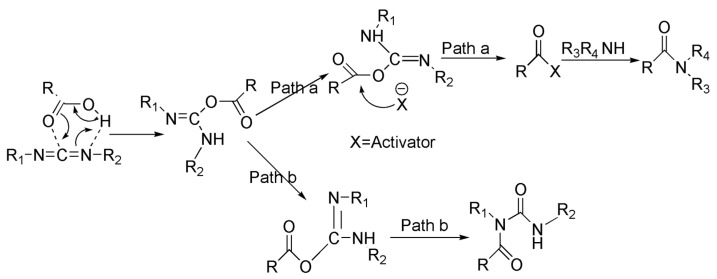
Diagram of the Synthetic reaction of macamides.

**Figure 3:**
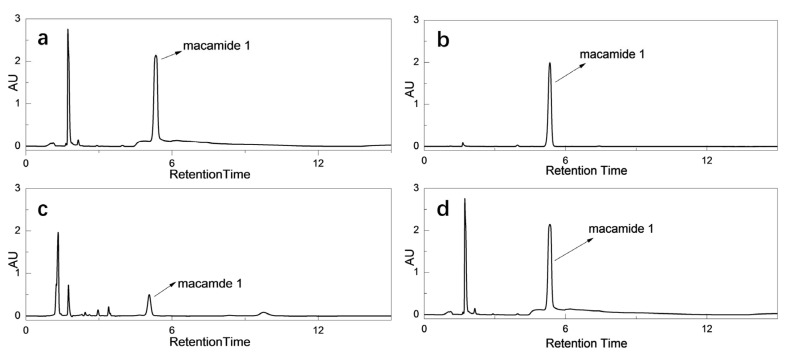
HPLC chromatograms of NBH samples: (**a**) the sample after acid-base reaction, (**b**) the sample after recrystallization, (**c**) the sample before the acid-base reaction, (**d**) the sample before recrystallization.

The macamide’s structural formulas and synthetic reaction diagram are presented in Figure 1 and Figure 2. Each macamide required different purification procedures, including an acid-base reaction, recrystallization, and silica gel column chromatography. The macamide purification method described herein is markedly different from the methods reported previously [17]. The results show that experiment 1 is comparable to experiment 5: the purity of **1** increased from 61.64% to 97.63%. To further investigate the effects of the acid-base reaction on the purity of **1**, the following experiment was carried out: *n-*hexane was washed with 10% sodium hydroxide and hydrochloric acid successively, and the lower aqueous layer was discarded. The *n-*hexane layer was washed again using the same steps as above, and it was then quantitatively analyzed by HPLC separately both before and after washing (Figure 3). This confirms that sodium hydroxide can reduce the impurity content (mainly fatty acids) and increase the macamide purity. This may be due to the fatty acid combining with sodium hydroxide, which could lead to decreased solubility of the impurities in *n-*hexane. In addition, alkaline substances in the reaction mixture, including triethylamine, were also easily removed by hydrochloric acid. Macamides are alkaloid amides which can combine with hydrochloric acid to form unstable compounds. When this compound dissolves in *n-*hexane, it can be cleaved into macamides. Macamides would easily dissolve in *n-*hexane. Thus, the acid-base reaction enhances macamide purity.

**Figure 4:**
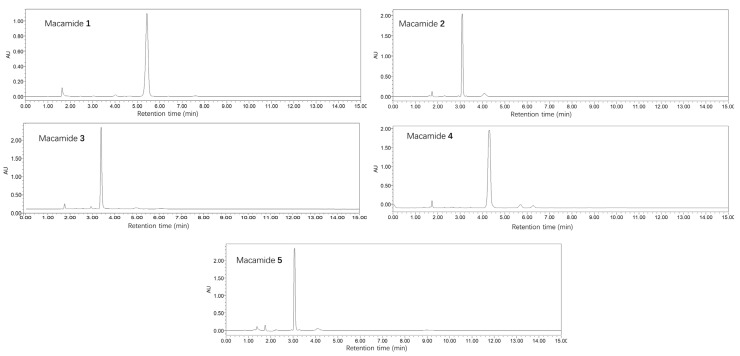
HPLC chromatograms of macamides **1**–**5**.

**Figure 5:**
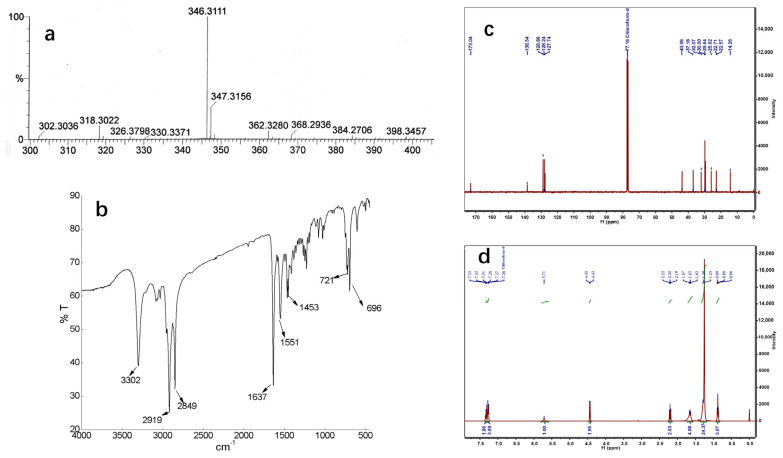
Characterization spectrums of NBH. (**a**) Mass spectrum of NBH, (**b**) Infrared spectrum of NBH, (**c**) ^13^C NMR spectrum of NBH, (**d**) ^1^H NMR spectrum of NBH.

The pure precipitates were analyzed by HPLC-DAD, ESI-MS, IR, and NMR (^1^H and ^13^C). Macamides **1**–**5** were detected by HPLC-DAD, with the highest peak corresponding to the respective macamide (Figure 4). These macamide peaks accounted for more than 95% of the total. The molecular formulas of these compounds were determined to be C_23_H_39_NO, C_25_H_37_NO, C_26_H_41_NO_2_, C_25_H_39_NO, and C_26_H_39_NO_2_, respectively, using ESI-MS (Figure 5a and Figure S1). The UV spectra of compounds **1**–**5** showed chromophores for a benzyl group (λ_max_ 210 nm), and the IR spectra revealed absorptions at ν_max_ 3302 cm^−1^ for N-H and ν_max_ 1453 cm^−1^ for a carbonyl group (Figure 5b and Figure S2). The ^13^C NMR spectra exhibited an amide carbonyl peak (δ_C_ 173.13) and peaks corresponding to monosubstituted or polysubstituted benzene rings, as well as two or three disubstituted double bonds (δ_C_ 131.99, 130.27, 129.12, 128.44, 127.63, 126.96; each d; C-9, C-10, C-12, C-13, C-15, C-16, respectively) (Figure 5c and Figure S3). The ^1^H NMR spectrum (Figure 5d and Figure S4) exhibited two or three *cis*-coupled olefinic protons at δ 5.37 (d, *J* = 4.0 Hz, H-9) and 5.36 (m, H-10), four other olefinic protons between δ 5.31 and 5.34 (m, H-12, H-13, H-15, and H-16), a primary methyl group at δ 0.87 (t, *J* = 4.0, 8.0, H-16) or δ 0.97 (t, *J* = 4.0, 8.0, H-18), as well as other protons between δ 1.30 and 1.33, assigned to the methylene group. Compared with macamide **1** (NBH), the other four compounds (**2**–**5**) contain two or three unsaturated double bonds and a 6-substituted phenyl ring replacing the methoxy group, which is verified in IR, MS, ^1^H, and ^13^C NMR spectra (Supplementary data).

### 2.2. Anti-Fatigue Activity of NBH

#### 2.2.1. Forced Swimming Test

**Figure 6:**
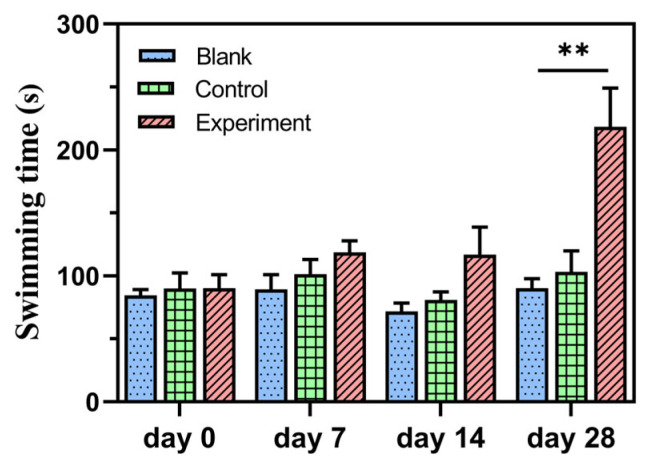
The effects of NBH on the forced swimming time to exhaustion in mice. The data were expressed as means ± SD, with n = 6. The double asterisk ** indicates a highly significant difference (*p* < 0.01) compared to the blank group.

It is widely accepted that exercise endurance is a direct and invariable factor in screening anti-fatigue agents, which is determined by the length of forced swimming time [9,18,19,20]. In this study, a forced swimming time (FST) model was developed to assess the fatigue-relieving effect of NBH by recording the swimming time of mice from the beginning of day 0; the swimming times of each group showed little difference. With constant NBH gavage, significant differences were observed in the NBH groups on day 28 (*p* < 0.05), and the increased ratio of swimming time was 105% (Figure 6). There was no significant difference between the control groups and the blank groups. The results showed that NBH played an important role in extending swimming time. Body weight was recorded on days 0, 7, 14, and 28. Compared to the normal increases, there were no significant differences in body weight changes between the two groups.

#### 2.2.2. Effect of NBH on Biochemical Paraments Related to Fatigue

The FST results demonstrate that NBH plays a significant role in fatigue activity. Therefore, the biochemical parameters related to fatigue were measured to investigate a possible mechanism. For example, liver glycogen (LG), blood urea nitrogen (BUN), lactate dehydrogenase (LDH), blood ammonia (BA), and blood lactic acid (BLA) were measured [21].

**Figure 7:**
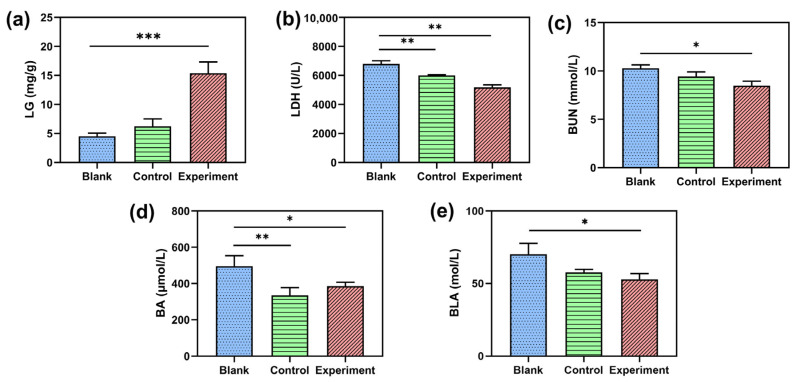
Effect of different supplementation on biochemical paraments in mice. (**a**): liver glycogen (LG); (**b**): lactic dehydrogenase (LDH); (**c**): blood urea nitrogen (BUN); (**d**): blood ammonia (BA); (**e**): blood lactic acid (BLA). (Data were expressed means ± SD, n = 6, *, *p* < 0.05 compared with that in the blank group; **, *p* < 0.01 compared with that in the blank group; ***, *p* < 0.001 compared with that in the blank group).

Energy depletion and deficiencies can lead to fatigue and reduce the body’s resistance. Liver glycogen (LG) is energy for exercise which is derived initially from the liver and converts lactate back to glycogen and releases glycogen into the blood [20]. Therefore, an increase in liver glycogen indicates enhanced exercise capacity and endurance capacity. The experimental results show that the content of the NBH group was significantly higher than that of the blank group, which was 241% (Figure 7a).

BUN is another indicator of fatigue in the body. It is the metabolic outcome of protein and amino acids when the body suffers from exhaustive exercise, which is derived initially from the liver and later excreted out of the body through the kidneys [22]. In brief, the more exercise the body undergoes, the more vitally the BUN level increases. As shown in (Figure 7b), BUN levels in the NBH group were significantly reduced by 18% (*p* < 0.05). The experimental results show that NBH can effectively reduce the accumulated BUN. LDH is an enzyme that plays an important role in eliminating lactic acid and providing energy under anaerobic conditions [23]; in other words, the more severe the muscle damage, the higher the serum levels of LDH [24]. The activities of LDH in the control group and NBH group were significantly lower than that of the blank group, by 11% (*p* < 0.01) and 23% (*p* < 0.01), respectively (Figure 7c). This experiment shows that NBH can relieve fatigue caused by muscle damage. BA is a metabolite produced by the breakdown of amino acids in the body, which is translated into urea in the liver. The level of BA that was raised may be associated with the body’s fatigue and liver glycogen decrease [25]. Compared with the blank group, the control group and NBH group showed significant differences, with the content of BA decreased by 30% (*p* < 0.01) and 21% (*p* < 0.05), respectively (Figure 7d). BLA, an important glycolysis product, is known to accumulate during intensive prolonged exercise [26]. In addition, with the repeated accumulation of lactic acid, there is eventually a decline of power output, leading to impairment of whole-body exercise performance; that is, fatigue develops. As shown in Figure 7e, the NBH group decreased the BLA content by 24% (*p* < 0.05) lower than the blank group.

In summary, five fatigue-related biochemical markers were examined to assess NBH anti-fatigue activity. The findings imply that NBH could improve mice’s endurance ability by raising the LG level and lowering the levels of BUN, LDH, BA, and BLA. For all four measures except LG, there were significant changes between the experimental group and the control group. These results demonstrated the anti-fatigue activity of NBH.

## 3. Discussion

A general method for amide synthesis involves the activation of a carboxylic fatty acid moiety with carbodiimide condensation agents (CCAs), followed by a reaction with an amine to generate the corresponding amide. CCAs, including *N*,*N*′-dicyclohexylcarbodiimide, *N*,*N*′-diisopropylcarbodiimide, and 1-(3-Dimethylaminopropyl)-3-ethylcarbodiimide hydrochloride (EDAC), are widely used in amide synthesis [27,28]. However, EDAC was chosen since it contributes to macamide purification. Subsequently, an acylation catalyst such as 4-dimethylaminopyridine (DMAP) or HOBt·H_2_O was selected for addition. The main reason for this addition was that the intermediates formed by nucleophilic substitution reactions of CCAs were not stable, which led to rearrangement byproducts. Previous studies have been conducted on the synthesis of macamides. For example, *N-*benzyl-15Z-tetracosenamide was rapidly synthesized using *cis*-15-tetracosenoic acid, DMAP, and dicyclohexyl carbodiimide [29] but had low product yield and purity. Another synthetic method using palmitic acid, 1′-carbonyldiimdazole, and DMAP afforded *N-*benzylpalmitamide in large quantities [16]. However, product purification requires silica gel column chromatography, which is complicated and inefficient. A previous paper [30] described a purification method in which 10% sodium hydroxide and hydrochloric acid were added to the reaction solution, followed by stirring and discarding the lower aqueous layer. This resulted in a product NBH with higher yield and purity. However, this method could not be applied on an industrial scale because of the high cost of the reaction raw materials. Hence, the synthetic method should take into consideration the commercial availability of raw materials, the content and purity of products, and reaction time. The purification process is the key point of synthetic reactions, as it is used for removing byproducts, the remaining materials, and other impurities. In the present study, the reaction materials, including EDAC, HOBt·H_2_O, triethylamine, and reaction byproducts, were water-soluble substances. Thus, extraction using organic solvents could easily reduce impurities and enrich the final product. For further purification, the acid-base reaction and recrystallization in the purification process first came up to increase product purity.

The effects of EDAC, HOBt·H_2_O, triethylamine, and the acid-base reaction on the yield and purity of NBH (**1**) are shown in Table 1. These results show that under the same conditions, experiment 1 produces comparable results with experiments 2–4. The yield of **1** in experiment 1 is significantly higher than those without treatment with EDAC, HOBt·H_2_O, and triethylamine. This is because EDAC, as a CCA, activates the carboxyl moiety of palmitic acid to form an intermediate by nucleophilic substitution. (Please see Supplementary Materials).

Because the intermediate is unstable, the use of HOBt as an acylation catalyst can keep the intermediate stable and stop it from generating related byproducts. In addition, using triethylamine as an acid-binding agent could accelerate the acylation reaction, which might mean that triethylamine could significantly improve the solubility of EDAC in DCM. In conclusion, EDAC, HOBt·H_2_O, and triethylamine are necessary to obtain macamides and enhance their yield and purity. Macamides are easily recrystallized from *n-*hexane due to their poor solubility at low temperatures when compared with impurities. Based on the above inference, recrystallization was utilized to increase the product purity. The recrystallization conditions are at 4 °C. Finally, each layer in the experimental section was analyzed by HPLC-UV, both before and after recrystallization. These results indicate that the low-polar impurities remained in the solution, and the macamides were easily separated. Due to their special physical properties, Macamides **2** and **4** should be recrystallized at −20 °C. However, macamides **3** and **5** were difficult to recrystallize, thus necessitating purification using silica gel column chromatography. Thus, after the acid-base reaction, many impurities in the precipitate were removed, and the macamides could be easily afforded by silica gel column chromatography.

FST is a reliable and valid model for evaluating anti-fatigue activity in mice, as several studies have reported a strong correlation between exercise endurance and the length of forced swim time [31]. Other biochemical parameters, such as muscle glycogen (MG), Adenosine Triphosphate (ATP), Adenosine Monophosphate (AMP), Methylenedioxyamphetamine (MDA), Superoxide Dismutase (SOD), have also been utilized to assess fatigue and the anti-fatigue effects of various supplements [32,33]. These parameters can provide additional insights into the mechanism of action of macamides. For example, increasing levels of ATP, AMP, and muscle glycogen have been linked to improved endurance, while reducing the levels of MDA and SOD has been suggested to decrease fatigue and enhance fatigue resistance. Additionally, 5-HT is a major biochemical marker for assessing fatigue in different species, and its levels can be detected by ELISA and HPLC with electrochemical and fluorescence detection [34]. The results of this study showed that NBH extended swimming time in test mice, increased levels of LG and reduced levels of BUN, LDH, BA, and BLA, indicating its potential for decreasing physical fatigue and improving exercise capacity. We have demonstrated that NBH can effectively improve mice’s endurance ability by altering several fatigue-related biochemical markers. However, it is important to note that the mechanism underlying this effect remains unclear. Future experiments could utilize techniques such as transcriptomics or metabolomics to gain a more comprehensive understanding of the molecular pathways involved in macamide’s anti-fatigue activity. Unsaturated fatty acids are important components of macamides. The transportation, metabolism, and action of unsaturated fatty acids in the body are complicated. It is well known that the degree of unsaturation can influence their biological activities. Studies have shown that highly unsaturated fatty acids can exhibit potent anti-inflammatory and antioxidant effects, such as eicosapentaenoic acid (EPA) and docosahexaenoic acid (DHA) [35]. There are also different reports indicating that DHA and EPA play an important role in alleviating exercise fatigue. However, the administration of DHA/EPA-triglyceride (TG) and DHA-phospholipid (PL) rather than DHA-TG can significantly extend the time and distance to running exhaustion in mice [36]. Future studies could focus on this aspect to determine if there is a correlation between macamide unsaturation and anti-fatigue activity.

## 4. Materials and Methods

### 4.1. Materials and Chemicals

All solvents and reagents were commercially available. Palmitic acid (AR, 99.0% pure), linoleic acid (99.0% pure), linolenic acid (99.0% pure), 3-methoxybenzylamine (98% pure), benzylamine (99.0% pure), HOBt·H_2_O (97% pure), EDAC (98.0% pure), and triethylamine (AR, 99.0% pure) were purchased from Aladdin (Shanghai, China). HPLC grade acetonitrile, *n-*hexane, hydrochloric acid, sodium hydroxide, and dichloromethane (DCM) (used as analytical reagents for the purification of macamides) were purchased from Wenrui (Guangzhou, Guangdong, China). Ultrapure water (18.2 MΩ cm at 25 °C) (MilliQ Millipore, Billerica, MA, USA) was used for HPLC analysis and synthesis of macamides.

### 4.2. Instrumental and Chromatographic Conditions

HPLC analysis was carried out on a Waters Alliance 2695 HPLC system (Waters, Milford, MA, USA), which was equipped with an inline vacuum degasser, a quaternary gradient pump, an autosampler, a DAD (2998) detector, and a Waters SunFire^TU^ C18 column (4.6 × 250 mm, 5 μm) and a Waters SunFire^TU^ C18 column with a flow rate of 1 mL/min for 15 min. The isocratic elution system comprised acetonitrile (95%) and water (5%). The DAD detection wavelength was set as 210 nm. Gradient elution chromatography was applied to determine the macamides extract from the maca. The solvent system consisted of (A) distilled water and (B) acetonitrile, using a gradient of 50:50 to 5:95 in 25 min, and the UV detection wavelength was 210 nm. The sample volume was 10 µL, and the flow rate was 1 mL/min. The injection volume of all samples was 10 µL. The optimal MS parameters were as follows: positive ion mode for MS detection; desolvation gas (N_2_) flow, 50 L/h; source voltage, 3 kV; transmission voltage, 30 V; all processes were controlled by Masslynx 4.1 software. In the HPLC-MS method, a Waters Acquity UPLC/Q-TOF Micro MS system equipped with an electrospray ionization (ESI) interface, working in positive ion mode, was used. Data processing was performed using Waters Masslynx 4.1 software. ^1^H and ^13^C NMR spectra were recorded on a Bruker Avance III instrument (Bruker, Fällanden, Switzerland) at 400 MHz (^1^H) and 100 MHz (^13^C) in CDCl_3_, using the residual solvent signal as an internal standard. IR spectra were recorded on a Spectrum 100 FT-IR spectrometer (PerkinElmer, Rodgau, Germany).

### 4.3. The synthesis of Five Main Macamides

#### 4.3.1. Preparation of NBH (**1**)

A DCM solution (50 mL) containing HOBt·H_2_O (103 mg, 0.76 mmol), EDAC (146 mg, 0.76 mmol), and palmitic acid (195 mg, 0.76 mmol) was stirred at 24 °C for 20 h. A solution of benzylamine (83 μL, 0.76 mmol) and triethylamine (264 μL, 1.5 mmol) dissolved in DCM (10 mL) was then added to the reaction mixture. After 4 h, the final solution was evaporated to dryness. Subsequently, 10% hydrochloric acid (10 mL) and *n-*hexane (50 mL) were added and stirred under ultrasonic conditions (200 W) for 5 min. The *n-*hexane layer was washed successively with 10% sodium hydroxide and 10% hydrochloric acid (10 mL), and the lower aqueous layers were discarded. Finally, the *n-*hexane solution was cooled to 4 °C and allowed to recrystallize for 24 h. The recrystallized precipitate (39.16% yield, 97.63% pure) was obtained through vacuum filtration as a white powder and dried. IR ν_max_ 3302, 2919, 2849, 1627, 1551, 1453, 721, 696 cm^−1^; ^1^H NMR (400 MHz, CDCl_3_) and ^13^C NMR (100 MHz, CDCl_3_) spectroscopic data of compound **1**–**5** were shown in Tables S1 and S2; MS, C_23_H_39_NO, [M + H]: 346.31. The spectroscopic data for the precipitate (MS, ^1^H, and ^13^C NMR) correspond to the data previously published for NBH.

#### 4.3.2. Preparation of *N*-Benzyl-9Z,12Z,15Z-octadecatrienamide (**2**)

Linolenic acid (233 μL, 0.76 mol, 99.0% pure), HOBt·H_2_O (103 mg, 0.76 mmol), and EDAC (146 mg, 0.76 mmol) were dissolved in DCM (50 mL). The reaction mixture was stirred at 25 °C for 20 h. Subsequently, benzylamine (83 μL, 99.0% pure) and triethylamine (264 μL, 1.5 mmol, 99.0% pure) were added to the resulting mixture. The same operating steps were followed for the preparation of NBH, except that the recrystallization temperature was −20 °C. Finally, the crystals were dried to yield the product as a brown powder (37.27% yield, 95.71% pure). IR ν_max_ 3298, 2929, 2856, 1648, 1544, 1455, 730, 699 cm^−1^; MS, C_25_H_37_NO, [M + H]: 368.30.

#### 4.3.3. Preparation of *N*-(3-Methoxybenzyl)-9Z,12Z-octadecadienamide (**3**)

Linoleic acid (236 μL, 0.76 mol; 99.0% pure), HOBt·H_2_O (103 mg, 0.76 mmol), and EDAC (146 mg, 0.76 mmol) were dissolved in DCM (50 mL). The reaction mixture was stirred at 24 °C for 20 h. Subsequently, 3-methoxybenzylamine (98 μL, 98.0% pure) and triethylamine (264 μL, 1.5 mmol, 99.0% pure) were added to the resulting mixture. The same operating steps were followed as for the preparation of NBH, except that the *n-*hexane solution was subjected to silica gel column chromatography (petroleum ether/ethyl acetate, 1:1) to yield the pure product as a colorless oil after solvent evaporation (31.25% yield, 96.21% pure). IR ν_max_ 3288, 3008, 2928, 2855, 1646, 1254, 1154, 1052, 737, 694 cm^−1^; MS, C_26_H_41_NO_2_, [M + H]: 400.32.

#### 4.3.4. Preparation of *N*-Benzyl-9Z,12Z-octadecadienamide (**4**)

Linoleic acid (236 μL, 0.76 mol; 99.0% pure), HOBt·H_2_O (103 mg, 0.76 mmol), and EDAC (146 mg, 0.76 mmol) were dissolved in DCM (50 mL). The reaction mixture was stirred at 24 °C for 20 h. Subsequently, benzylamine (83 μL, 99.0% pure) and triethylamine (264 μL, 1.5 mmol, 99.0% pure) were added to the resulting mixture. The same operating steps were followed for the preparation of NBH, except that the recrystallization temperature was −20 °C. Finally, the crystals were dried to yield the product as white oil (43.97% yield, 95.13% pure). IR ν_max_ 3288, 3009, 2926, 2855, 1645, 1549, 1455, 1252, 1029, 726, 697 cm^−1^; MS, C_25_H_39_NO, [M + H]: 372.31.

#### 4.3.5. Preparation of *N*-(3-Methoxybenzyl)-9Z,12Z,15Z-octadecatrienamide (**5**)

Linolenic acid (233 μL, 0.76 mol; 99.0% pure), HOBt·H_2_O (103 mg, 0.76 mmol), and EDAC (146 mg, 0.76 mmol) were dissolved in DCM (50 mL). The reaction mixture was stirred at 24 °C for 20 h. Subsequently, 3-methoxybenzylamine (98 μL, 98.0% pure) and triethylamine (264 μL, 1.5mmol, 99.0% pure) were added to the resulting mixture. The same operating steps were followed as for the preparation of NBH, except that the *n-*hexane solution was subjected to silica gel column chromatography (petroleum ether/ethyl acetate, 1:1) to yield the pure product as a light brown oil after solvent evaporation (30.79% yield, 95.92% pure). IR ν_max_ 3300, 2931, 2856, 1647, 1265, 1154, 1051, 700 cm^−1^; MS, C_26_H_39_NO_2_, [M + H]: 398.31.

### 4.4. The Preparation of Macamides-Rich Extracts

The dried maca powder (40 mesh) 1 g was mixed with diethyl ether 30 mL and then ultrasonic extracted for 30 min. The solution was centrifuged at 4000 rpm for 15 min, and then the supernatant was concentrated by a rotary vacuum evaporator to obtain a dry extract. A certain amount of diethyl ether extract of maca was further washed with ethanol 5 mL and 5 mL sodium hydroxide (1 mol/L) in a water bath at 50 °C for 50 min, and 2 mL carbonic acid was added to neutralize. Finally, the concentrated solution was dissolved with *n-*hexane 5 mL and water 30 mL by ultrasonic to obtain *n-*hexane content. Finally, the *n-*hexane layer was dried, and its macamide content was analyzed using HPLC [37,38].

### 4.5. Anti-Fatigue Activity of NBH

#### 4.5.1. Animals and Groups

The following anti-fatigue activity experiment was designed mainly based on previous research [19,39,40]. Adult male C57 mice, weighing an average of 20 g, were obtained from Guangdong Medical Laboratory Animal Center (certificate number SCXK-2013-0002). The animals were housed under controlled conditions (room temperature of 24 °C, relative humidity of 38–60%,12 h/12 h light/dark cycle) and had free access to food and water. All experiments were performed by the Animal Care and Use Committee of Guangdong Pharmaceutical University.

Before the experimental mice were forced to swim, the mice that failed to learn to swim were eliminated. After that, 18 mice were randomly divided into three groups: (1) blank group, treated with distilled water; (2) control group: treated with 1000 mg/kg extract of maca; (3) experiment group (NBH): treated with 10 mg/kg. Oral gavage was performed at 2 p.m. for 28 days.

#### 4.5.2. Forced Swimming Times and Biochemical Paraments

During 28 days of gavage, the body weight of each group of mice was measured before being forced to swim on days 0, 7, 14, and 28. The swimming test was as follows: each time mice had been orally gavaged, they were allowed to rest for 30 min and loaded with a tin wire (10% of body weight) attached to their tails. Then, the mice were individually dropped into a plastic swimming pool (50 cm × 50 cm × 40 cm) containing 35 cm deep water at 25 ± 1 °C. The exhaustive swimming time was immediately recorded when mice failed to rise to the surface of the water to breathe within 3 s. After the forced swimming test, the mice were allowed to rest for 30 min and then were anesthetized with ether. Blood samples were collected from the eyeball and centrifugated at 4000 rpm for 10 min, which was used for BUN, BLA, LDH, and BA determination. After the blood was obtained, the liver was immediately taken out and analyzed for HG. All kits were purchased from Nanjing Jiancheng Bioengineering Institute (Nanjing, China).

### 4.6. Statistical Analysis

Experimental data were represented as mean ± standard deviation. Differences between groups were estimated by one-way analysis of variance (ANOVA) using GraphPad Prism and Excel. Differences at *p* < 0.05 or *p* < 0.01 were considered significant.

## 5. Conclusions

We have successfully developed an efficient and simple synthetic protocol for the synthesis of the five main macamides, achieving a purity of over 95% and a yield of more than 30%. The macamides were identified using a combination of HPLC, MS, ^1^H and ^13^C NMR, and IR techniques. By synthesizing macamides in large quantities rather than using maca extracts, they can be utilized in pharmacological experiments and for quality control of maca products. Furthermore, NBH is the representative monomer in macamides. Animal experiments demonstrated that it has anti-fatigue activity, which can be primarily attributed to its ability to raise the LG level and reduce the levels of BUN, LDH, BA, and BLA. NBH may be a promising candidate for alleviating fatigue for further study.

## Figures and Tables

**Table 1:** Effects of EDAC, HOBt, triethylamine, and acid-base reactions on the yield and purity of macamide.

No.	EDAC	HOBt·H_2_O	Triethylamine	Acid-Base Reaction	Precipitate Yield (%)	Purity (%)
1	Y *	Y	Y	Y	39.16	97.63
2	N **	Y	Y	Y	0	0
3	Y	N	Y	Y	0	0
4	Y	Y	N	Y	100.37	13.21
5	Y	Y	Y	N	64.25	61.64

* Y: addition; ** N: no addition.

## Data Availability

Data sharing is not applicable to this article.

## Supplementary Materials

The following supporting information can be downloaded at: https://www.mdpi.com/article/10.3390/molecules28093943/s1, Table S1: ^1^H NMR spectrums data of macamides **1**–**5**; Table S2: ^13^C NMR spectrums data of macamides **1**–**5**; Figure S1: Mass spectrums of macamides **2**–**5**; Figure S2: Infrared spectrums of macamides **2**–**5**; Figure S3: ^13^C NMR spectrums of macamides **2**–**5**; Figure S4: ^1^H NMR spectrums of macamides **2**–**5**.

## Sample Availability

Samples of the compounds Macamides **1**–**5** are available from the authors.
