# Supplementary material for: The Efficient Synthesis and Anti-Fatigue Activity Evaluation of Macamides: The Unique Bioactive Compounds in Maca
Source: Molecules. 2023 May 7;28(9):3943. doi: 10.3390/molecules28093943 (PMC10180231; doi:10.3390/molecules28093943)
Supplement: Supplementary file 1 [file molecules-28-03943-s001.zip › molecules-2378328-supplementary.pdf]

SUPPLEMENTARY MATERIALS

The efficient synthesis and anti-fatigue activity evaluation of macamides: the unique bioactive compounds in maca

Tao Liu [1], Ziyan Peng [1,2], Wei Lai [1,2], Yan Shao [1], Qing Gao [1,3], Miaoxin He [1,3], Wan Zhou [1,3], Lirong Guo [1,3], Xiaobao Jin [1], Jiyao Kang [4] and Hui Yin [1,2*]

1 Guangdong Provincial Key Laboratory of Pharmaceutical Bioactive Substances, School of Basic Medical Sciences, Guangdong Pharmaceutical University, Guangzhou 510006, China; liutao@gdpu.edu.cn (T.L.); 15812315359@163.com (Z.P.); laiwei1227@foxmail.com (W.L.); 2112240280@gdpu.edu.cn (M.H.)

2 Department of Microbiology and Immunology, Guangdong Pharmaceutical University, Guangzhou 510006, China

3 School of pharmacy; Guangdong Pharmaceutical University, Guangzhou 510006, China

4 National Key Laboratory of Biochemical Engineering, Institute of Process Engineering, Chinese Academy of Sciences, Beijing 100080, China; jykang@ipe.ac.cn

* Correspondence: huiyin0103@gdpu.edu.cn

Content

**Table S1.**	^{1}H NMR spectrums data of macamide 1-5	1
**Table S2.**	^{13}C NMR spectrum data of macamide 1-5	2
**Figure S1.**	Mass spectrums of macamide 2-5	3
**Figure S2.**	Infrared spectrums of macamide 2-5	4
**Figure S3.**	^{13}C NMR spectrums of macamide 2-5	5
**Figure S4.**	^{1}H NMR spectrums of macamide 2-5	6

Table S1. ^{1}H NMR spectrum data of macamide **1-5. Spectrums were recorded at 400 MHz in CDCl3.**

Protons	1	2	3	4	5
2	2.20 t (4.0, 8.0)	2.21 t (4.0, 8.0)	2.20 t (4.0, 8.0)	2.20 t (4.0, 8.0)	2.20 t (4.0, 8.0)
3	1.65 m	1.65 m	1.67 m	1.65 m	1.65 m
4	1.25 m	1.31 m	1.31 m	1.31 m	1.30 m
5	1.25 m	1.31 m	1.31 m	1.31 m	1.30 m
6	1.25 m	1.31 m	1.31 m	1.31 m	1.30 m
7	1.25 m	1.31 m	1.31 m	1.31 m	1.30 m
8	1.25 m	2.06 t (8.0, 8.0)	2.05 d (8.0)	2.05 d (8.0)	2.07 m
9	1.25 m	5.31 d (4.0)	5.39 m	5.41 m	5.37 d (4.0)
10	1.25 m	5.36 dd (20.0, 16.0)	5.37 m	5.39 m	5.36 m
11	1.25 m	2.80 t (8.0, 4.0)	2.76 t (4.0, 8.0)	2.77 t (4.0, 8.0)	2.80 t (8.0, 4.0)
12	1.25 m	5.32 m	5.35 m	5.35 m	5.34 m
13	1.25 m	5.33 m	5.34 m	5.32 m	5.32 m
14	1.25 m	2.80 t (8.0, 4.0)	2.04 d (8.0)	2.04 d (8.0)	2.80 t (8.0,4.0)
15	1.29 m	5.37 m	1.31 m	1.31 m	5.32 m
16	0.87 t (4.0, 8.0)	5.33 m	1.31 m	1.31 m	5.31 m
17	-	1.30 m	1.31 m	1.31 m	2.05 m
18	-	0.97 t (8.0, 8.0)	0.90 t (4.0, 4.0)	0.89 t (4.0, 8.0)	0.97 t (4.0, 8.0)
1'	4.44 d (8.0)	4.45 d (8.0)	4.40 d (8.0)	4.43 d (8.0)	4.40 d (8.0)
3'	7.21 m	7.28 d (4.0)	6.80 d (4.0)	7.28 m	6.86 m
4'	7.33 m	7.33 m	6.84 m	7.35 m	6.86 m
5'	7.28 m	7.32 m	6.81 m	7.29 m	6.82 m
6'	7.36 m	7.35 m	-	7.33 m	-
7'	7.31 m	7.29 d (4.0)	5.72 s	7.28 m	5.77 s
N-H	5.71 br s	5.70 br s	7.28 br m	5.71 br s	7.26 br m
O-CH_3	-	-	3.79 s		3.79 s

Table S2. ^{13}C NMR spectrum of macamide **1-5**. Spectrums were recorded at 100 MHz in CDCl3.

Carbon	1	2	3	4	5
1	173.04 s	173.38 s	173.07 s	172.85 s	173.03 s
2	37.18 t	37.27 t	36.91 t	36.84 t	36.83 t
3	25.92 t	29.90 t	26.06 t	25.70 t	29.83 t
4	29.64 t	29.74 t	29.74 t	27.34 t	29.15 t
5	30.00 t	29.70 t	29.40 t	29.84 t	29.71 t
6	30.00 t	29.58 t	27.18 t	29.84 t	29.61 t
7	30.00 t	27.66 t	26.06 t	29.84 t	27.40 t
8	30.00 t	25.52 t	26.02 t	29.84 t	25.25 t
9	30.00 t	132.34 d	128.18 d	130.45 d	131.99 d
10	30.00 t	128.19 d	113.59 d	127.45 d	130.27 d
11	30.00 t	26.21 t	22.61 t	22.74 t	25.25 t
12	30.00 t	128.14 d	113.08 d	127.45 t	129.12 d
13	30.00 t	127.98 d	128.00 d	130.15 d	128.44 d
14	32.07 t	26.08 t	27.18 t	29.84 t	25.25 t
15	22.71 t	127.72 d	26.06 t	29.84 t	127.63 d
16	14.26 q	130.98 d	31.62 d	31.80 d	126.96 d
17	-	20.89 t	26.02 t	25.88 t	20.28 t
18	-	14.54 q	14.31 q	14.18 q	14.79 q
1'	43.96 t	43.90 t	43.54 t	43.75t	43.81 t
2'	138.56 s	138.90 s	140.09 s	138.48 s	140.49 s
3'	128.24 d	129.06 d	130.56 d	128.90 d	120.17 d
4'	128.86 d	128.69 d	130.18 d	127.15 d	126.96 d
5'	127.74 d	128.69 d	120.31 d	127.15 d	113.41 d
6'	128.86 d	128.69 d	160.08 d	127.95 d	160.18 d
7'	128.24 d	129.06 d	129.75 d	128.90 d	113.01 d
O-CH_3	-	-	55.45 d		55.13 d

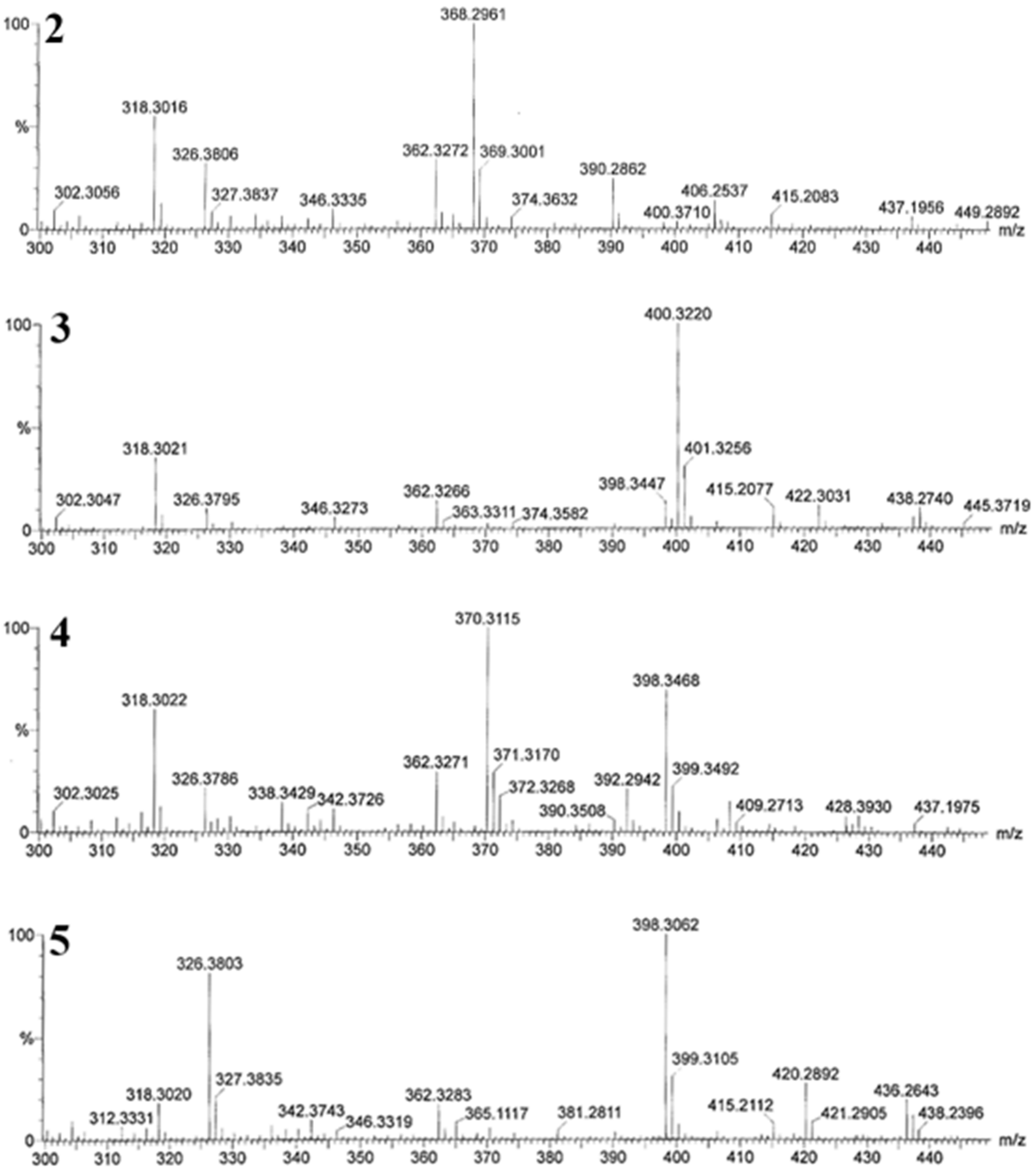

Figure S1. Mass spectrums of macamide **2-5.**

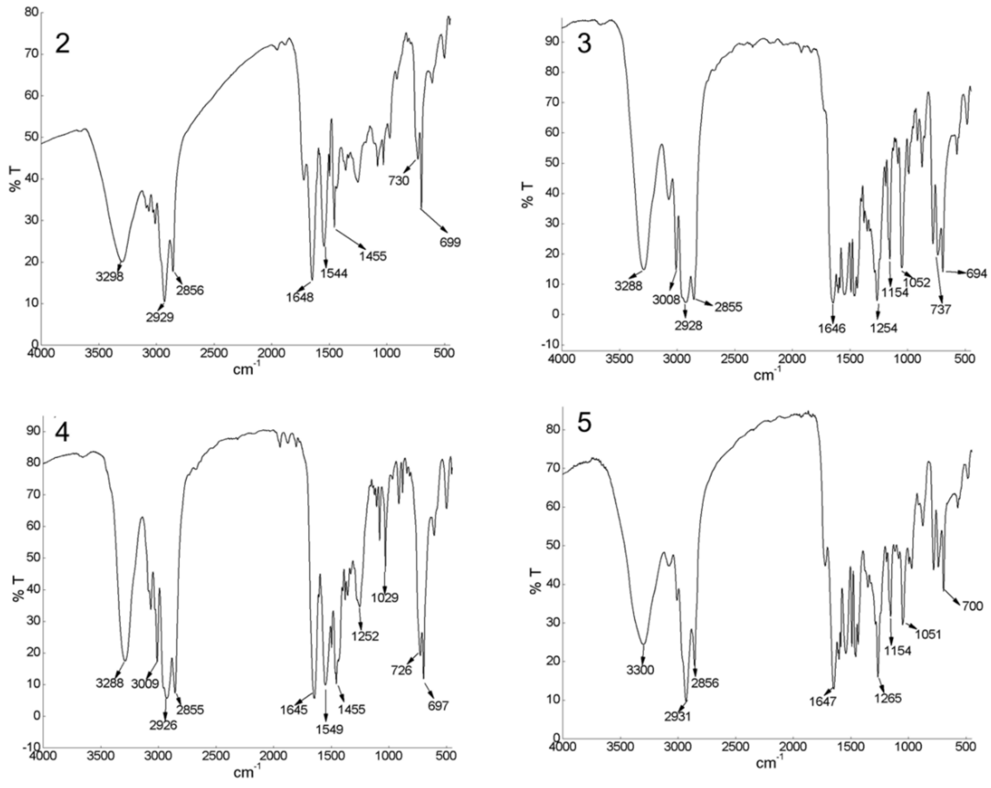

Figure S2. Infrared spectrums of macamide **2-5.**

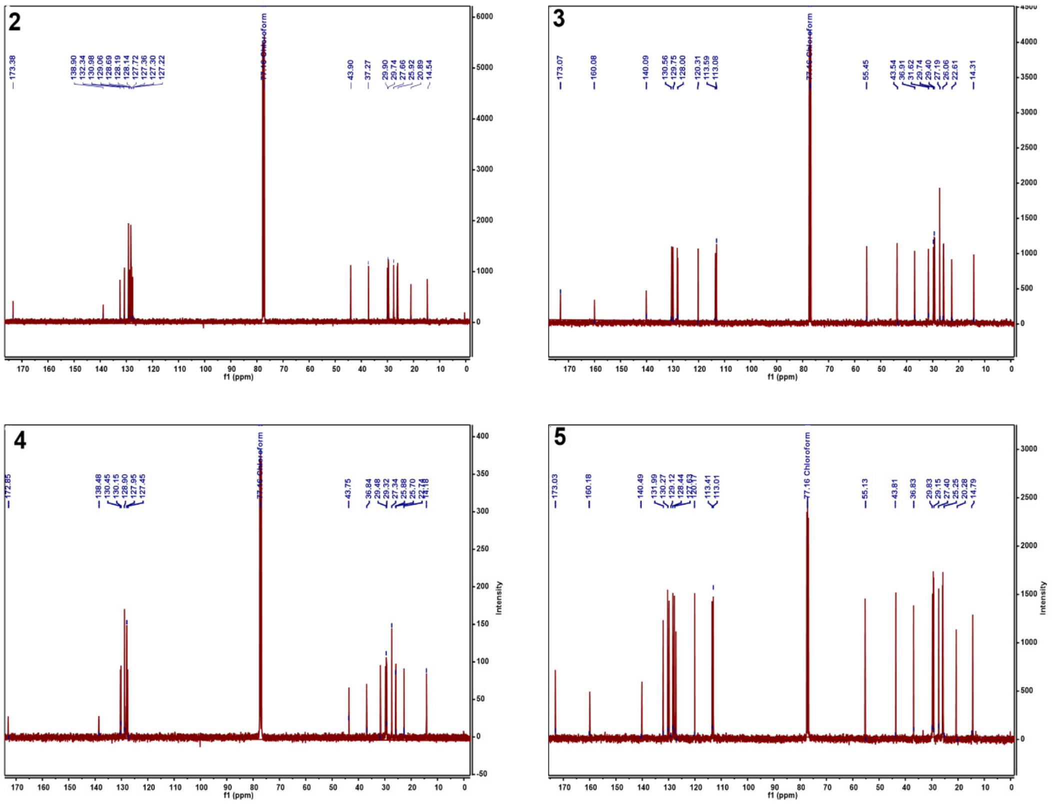

Figure S3. ^{13}C NMR spectrums of macamide **2-5.**

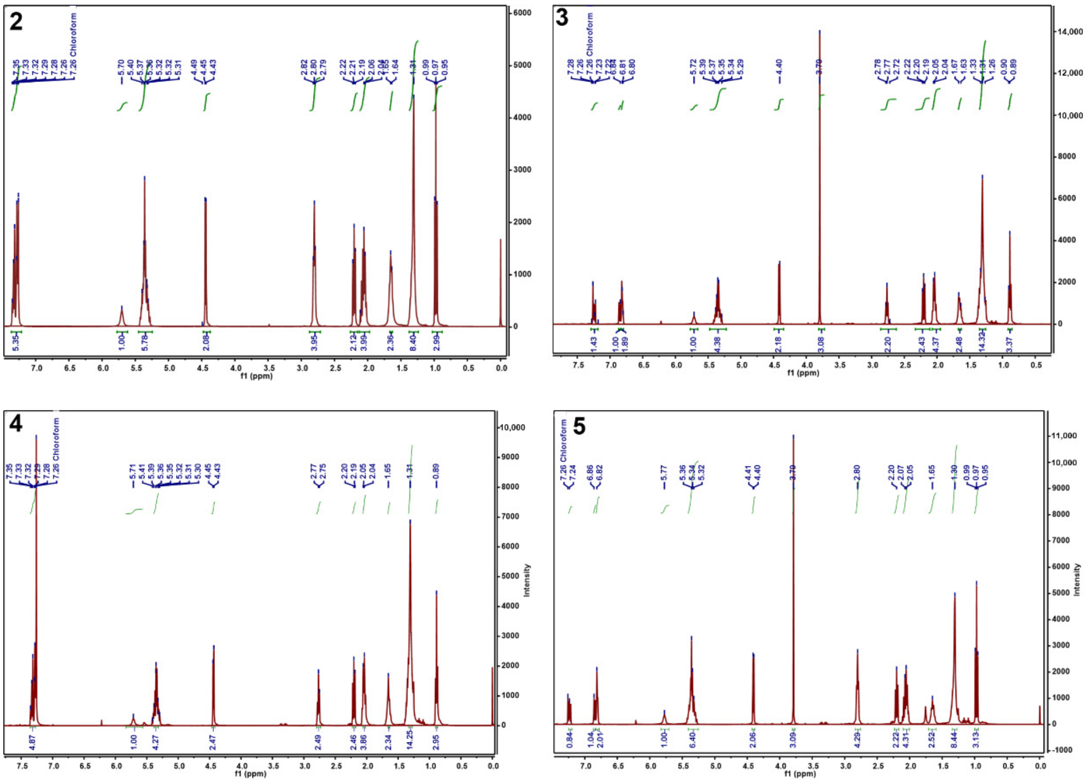

Figure S4. ^{1}H NMR spectrum of macamide **2-5.**
